# A Randomized Controlled Trial Study of a Multimodal Intervention vs. Cognitive Training to Foster Cognitive and Affective Health in Older Adults

**DOI:** 10.3389/fpsyg.2022.866613

**Published:** 2022-06-20

**Authors:** Maria Brasser, Sascha Frühholz, Andres R. Schneeberger, Gian G. Ruschetti, Rahel Schaerli, Michèle Häner, Barbara Studer-Luethi

**Affiliations:** ^1^Cognitive and Affective Neuroscience Unit, University of Zürich, Zürich, Switzerland; ^2^Department of Psychology, University of Oslo, Oslo, Norway; ^3^Department of Psychiatry, Psychotherapy and Psychosomatics, Psychiatric University Hospital, Zürich, Switzerland; ^4^Department of Psychology, University of Bern, Bern, Switzerland

**Keywords:** cognitive health, multimodal intervention, cognitive training, activity, prevention

## Abstract

Research over the past few decades has shown the positive influence that cognitive, social, and physical activities have on older adults’ cognitive and affective health. Especially interventions in health-related behaviors, such as cognitive activation, physical activity, social activity, nutrition, mindfulness, and creativity, have shown to be particularly beneficial. Whereas most intervention studies apply unimodal interventions, such as cognitive training (CT), this study investigates the potential to foster cognitive and affective health factors of older adults by means of an autonomy-supportive multimodal intervention (MMI). The intervention integrates everyday life recommendations for six evidence-based areas combined with psychoeducational information. This randomized controlled trial study compares the effects of a MMI and CT on those of a waiting control group (WCG) on cognitive and affective factors, everyday life memory performance, and activity in everyday life. Three groups, including a total of 119 adults aged 65–86 years, attended a 5- or 10-week intervention. Specifically, one group completed a 10-week MMI, the second group completed 5-week of computer-based CT followed by a 5-week MMI, whereas the third group paused before completing the MMI for the last 5 weeks. All participants completed online surveys and cognitive tests at three test points. The findings showed an increase in the number and variability of activities in the everyday lives of all participants. *Post hoc* analysis on cognitive performance of MMI to CT indicate similar (classic memory and attention) or better (working memory) effects. Furthermore, results on far transfer variables showed interesting trends in favor of the MMI, such as increased well-being and attitude toward the aging brain. Also, the MMI group showed the biggest perceived improvements out of all groups for all self-reported personal variables (memory in everyday life and stress). The results implicate a positive trend toward MMI on cognitive and affective factors of older adults. These tendencies show the potential of a multimodal approach compared to training a specific cognitive function. Moreover, the findings suggest that information about MMI motivates participants to increase activity variability and frequency in everyday life. Finally, the results could also have implications for the primary prevention of neurocognitive deficits and degenerative diseases.

## Introduction

Both the continuously increasing life expectancy and average age of humans are causing rising numbers of neurocognitive deficits and diseases in older people. Today, more than 55 million people suffer from dementia worldwide, and every year the number increases by 10 million (Alzheimer’s Association, 2022). In Switzerland, the Alzheimer Swiss association estimates that by 2050, 315,400 people are expected to become ill with dementia. Cognitive deficits are a strong predictor of impaired mental health and reduced well-being in old age, and have physical, psychological, and social impacts on the caretakers, families, and society at large (Alzheimer’s Disease International, 2019). Furthermore, middle-aged people are generally concerned about developing cognitive deficits in older age. The world Alzheimer report (2019), which included 70,000 people across 155 countries, reports that almost 80% of the general public is concerned about developing dementia. And about 30% believe that there is nothing they can do about it. Such worries may even eventually cause a decline in executive functioning (Kassem et al., 2017). These numbers point to the importance of researching intervention regimens designed to increase cognitive and affective health factors and thereby reduce cognitive impairments.

One approach shown to be particularly beneficial in promoting cognitive abilities and at preventing and delaying cognitive decline is cognitive training (CT; Kane et al., 2017). Research on functional CT has produced promising results in the last few decades, especially in connection with improved working memory performance, reaction time, and cognitive flexibility for people with dementia and for older adults (Jaeggi et al., 2010; Dresler et al., 2017; Zhang and Chignell, 2020). The most applied CT methods comprise computer-based stimulus-reaction tasks trained regularly over several weeks. CT focuses especially on working memory function, which is a core mechanism and high-order cognitive area (Ma et al., 2014) related to fluid intelligence, reasoning, and cognitive control (Redick and Engle, 2011; Unsworth et al., 2014). Park and Ingles (2001) proposed that different levels of CT transfer effects were dependent on the similarity of the involved process to the target outcome of a trained task. Thus, far transfer effects are measured by outcomes that do not rely on the same core process (*cf.* Hong et al., 2021). CT may even influence additional cognitive and affective outcomes, similar to how training the physical heart can achieve multiple beneficial health outcomes (Sartory et al., 2005; Owens et al., 2013; Jaeggi et al., 2021). However, besides research on, and commercial success of, especially computer-based CT, results regarding the extent of the effects of CT are inconsistent (see Bottiroli et al., 2013, for a review; Chein and Morrison, 2010; Redick et al., 2013). Most cognitive interventions significantly influence specific cognitive functions and some even indicate transfer to daily life in healthy older adults (Ball et al., 2002; Bottiroli et al., 2013; Tennstedt and Unverzagt, 2013). Although, results on further-reaching transfer effects (e.g., conclusive thinking, executive function, and well-being) are heterogeneous (Klingberg et al., 2002; Jaeggi et al., 2008; Noack et al., 2009; Chein and Morrison, 2010). Current meta-analysis determines that working memory training often has very specific effects, and only limited transfer effects to related competencies can be consistently demonstrated (Hudes et al., 2019; Roesch-Ely and Baum, 2019).

Besides cognitive intervention, other approaches exist to promote cognitive and affective health factors and prevent cognitive decline (Vemuri et al., 2014; Kuiper et al., 2015; Buchmann et al., 2019). First, an important approach seems *social activity*. Loneliness, isolation, and few social contacts are correlated with increased risk for cognitive decline and dementia (see Masi et al., 2011; Cacioppo et al., 2015, for a meta-analysis). Different studies on interventions designed to increase social activity (e.g., communication training) showed a positive influence on cognitive factors (Videler et al., 2020; e.g., Bambini et al., 2020; Christelis and Dobrescu, 2020, for a meta-analysis). Second, *physical health-related activities,* such as physical exercise or healthy food interventions, were shown to prevent cognitive decline (e.g., Mediterranean diet or physical activity goals; Abbott et al., 2004; Lautenschlager et al., 2008; Coelho et al., 2009; Audiffren et al., 2011; Morris et al., 2015; Schlaff et al., 2018). Also, positive effects of coordination training interventions or so-called “brain gym exercises” were reported for working memory performance and well-being in older male adults (Voelcker-Rehage et al., 2011; Tootak and Abedanzadeh, 2021). Third, *mindfulness training intervention s*howed improvements in cognition in older adults with and without cognitive impairment (Zhang et al., 2018; Wells et al., 2019), with additional effects on affective functions (e.g., increased positivity, a greater sense of coherence, more empathy, lower perceived stress, and cortisol level; Grossman et al., 2004; Vago and Silbersweig, 2012). Fourth, *creative activities*, such as arts, dance, expression, music, or storytelling interventions, were shown to be beneficial. Creativity was not only linked to physiological health indicators but moreover to improved cognitive functions (e.g., Sharma and Babu, 2017). Individuals who practiced more creative exercises demonstrated higher cognitive reserve in comparison to routine activities (Colombo et al., 2018). Creative therapy interventions with older adults with and without dementia also showed a greater positive effect on cognitive flexibility than the use of standard CT (Martinec and Lera, 2018; Zhao et al., 2018). Fifth, *humor*-related interventions showed a reduction of cortisol, decreased stress, and protection of cognitive abilities in older adults (*cf.* Mallya et al., 2018).

Most of the before-mentioned intervention studies focused mostly on single factors (e.g., only CT) and their impact on targeted functions. However, studies that combined different of these intervention factors produced promising results regarding transfer effects on non-trained factors, such as cognitive and executive functions, activities of daily living, and emotional coping (*cf.* for recent reviews Chalfont et al., 2020; Grueso and Viejo-Sobera, 2021; Hopkinson, 2021; Rundek et al., 2021; Omura and Araki, 2022). Other interventions with older people that combined CT with physical exercise or with diet showed effects on well-being and cognitive abilities in everyday life (Oswald et al., 2006; Ngandu et al., 2015; Maffei et al., 2017; Takeuchi et al., 2020). Also, a general physically, cognitively, and socially active lifestyle acts as a buffer for degenerative diseases and can reduce the risk for cognitive decline and dementia by around 40% (Livingston et al., 2020). Older people increasingly suffer from loneliness and isolation (WHO Guidelines, 2019), and the current situation with the COVID-2019-pandemic has resulted in more restrictions for possible stimulating activities (e.g., sports meetings, cultural events, etc.). Intervention regimens that guide and support middle-aged and older peoples’ daily cognitive activation and affective care are particularly important. However, there is still little knowledge about the effects of MMI, which include more than two or three intervention factors on cognitive and affective functions.

This study accordingly aimed to bolster cognitive and affective health factors in older adults and to prevent degenerative diseases with a MMI. Based on the latest meta-analysis, we combined six intervention areas shown to be particularly beneficial against cognitive decline (for a recent meta-analysis, see Astrup et al., 2019; WHO Guidelines, 2019; Sabbagh et al., 2022). Compared to the large research field of CT, a comprehensive cognitive and affective intervention approach offers further potential for research and for the elderly. This is the first study that measures the effects of MMI with six intervention areas in a longitudinal design in comparison to CT.

The MMI investigated here includes six categories of exercises connected to healthy cognitive aging: physical, cognitive, social, mindful, coordination, and creative activation. The intervention integrates everyday-life exercises and activities as well as lifestyle recommendations and adds psychoeducational information about each of the promoted areas. Generally, the intervention intended to encourage participants to be more active in everyday life. Finally, the intervention follows a self-determination theory approach for adult learning in applying the three motivational factors: autonomy, competence, and relatedness (Deci and Ryan, 2008). This MMI was already evaluated in a cross-sectional pilot study in 2020 with 660 individuals who took part in the intervention (made publicly available through the University of Bern, Switzerland) to support elderly people in cognitive activation and affective care in times of the COVID-19-pandemic (Studer-Luethi et al., 2021). The results demonstrated perceived benefits in memory, well-being, attitude toward the aging brain and lifestyle habits.

To compare a multimodal approach with a single-component intervention, we here compared the effects of the MMI in a longitudinal setup to those of a single-modal CT and to a WCG. For the CT group, we used a computer-based CT program. The CT had already been evaluated several times and was used in various studies with different population groups (Bernhardt et al., 2002; Jaeggi et al., 2010; Gajewski and Falkenstein, 2012; Dresler et al., 2017; Fellmann et al., 2017; Fuermaier et al., 2017; Soveri et al., 2017a,b; Frankenmolen et al., 2018; Thoma, 2019).

We chose a three-test-point-design where the intervention conditions differed for all groups only between time point 1 (T1) and time point 2 (T2), while between T2 and time point 3 (T3) all groups received the same MMI. This design was chosen for two reasons. First, all participants could complete and hopefully benefit from the MMI at least once during the study. Second, the design allowed randomization, single-blind procedures, and comparison between the different intervention regimens. There is no existing standard for measures of cognitive decline prevention, so we used several recommended cognitive tests from a European task force consensus (Vellas et al., 2008).

We hypothesized that the interventions would increase cognitive performance (e.g., working memory, attention, and fluid intelligence) for the MMI-and the CT group. Moreover, we expected that the MMI would show further-reaching transfer effects on the number and variability of activities in everyday life, memory performance in everyday life, as well as on perceived benefits in well-being, stress, and attitude toward the aging brain.

## Materials and Methods

### Participants and Study Design

This randomized controlled single-blind trial study compared the effects of a multimodal intervention (MMI) to those of cognitive training (CT) and a waiting control group (WCG). Study participation was publicly offered to interested people between 65 and 90 years old in the German-speaking part of Switzerland with access to a computer (incl. email) and a printer. They were recruited with flyers and newsletters in retirement homes, senior universities, medical practices, and *via* social media channels, friends, and acquaintances from the personal environment of the study leaders. One-hundred thirty one participants (43 male, 88 female) were recruited between March and August in 2021. Demographic data comprised age (*M* = 71.54, *SD* = 5.22, age range: 65–86), years of education [primary school = 1 (0.84%), lower secondary school = 1 (0.84%), apprenticeship = 36 (30.25%), high school = 11 (9.24%), Bachelor = 42 (35.29%), Master or higher = 28 (23.53%)], sex (67.18% female), health condition [51 (42.86%) very good, 65 (54.62%) rather good, 3 (2.52%) rather bad, and 0 (0.00%) bad], and living situation [34 (28.57%) living alone, 75 (63.03%) living with someone, 5 (4.20%) living in the same house with other family members (e.g., children and grandchildren), and 5 (4.20%) living in the close distance of other family members].

**Figure 1:**
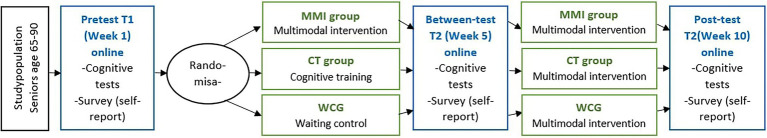
Visualization of the study design. All participants completed repetitive surveys and cognitive tests three times (Pre-test, Between-test, and Post-test). After randomization, they were assigned to three groups, the multimodal intervention (MMI) group, the cognitive training (CT) group, and the waiting control group (WCG). After 5 weeks, they all received the MMI for five more weeks.

All participants completed repetitive surveys and cognitive tests of about 60 min at three time-points, with 5 weeks of intervention time between time points. The groups had different intervention conditions between T1 and T2, but between T2 and T3, all participants completed the same MMI (Figure 1). Participation time for both interventions was about 60 min of activity per week. Research has shown that it cannot be assumed that CT intervention phases below 4 weeks lead to continuous effects (*cf.* Buschkuehl et al., 2012; Jaeggi et al., 2017; Biel et al., 2020). Therefore, we selected a short but still promising intervention phase length (5 weeks) and number of training units (three times a week). Both intervention groups had the possibility to connect and interact with other participants of the same group *via* an experience exchange group of six people on an online group chat or *via* a partner tandem on the telephone.

The participants were not paid and provided informed, written consent before participation, and the Ethical committee of the Swiss Canton of Bern approved the study (Code 2021-09-00001). The sample size was estimated based on prospective statistical assumptions compared with literature on similar CT interventions in similar settings (Roesch-Ely and Baum, 2019). We therefore assumed an improvement of at least eight points in memory performance (*T*-value scale from 20 to 80 points) and a SD of 4.5. With a significance level of *p* < 0.05 (the hypothesis was directional) and power of 0.8, the number of people per group was 27 participants. As research experience showed that CT studies show high drop-out rates (Roesch-Ely and Baum, 2019), and the intervention was expected to be intense, the number of subjects per group was increased to 45.

In our final sample, 131 individuals participated in the study with 119 completing the first survey and tests allowing their further inclusion in the study. Out of the 41 participants (mean age = 71.07 years; SD = 5.48; 27 male) originally assigned to the MMI group, 35 completed the MMI after the first 5 weeks and 32 completed the MMI after 10 weeks. Out of the 37 participants (mean age = 71.49 years; *SD* = 4.52, 23 male) originally assigned to the CT group, 23 participants completed the CT after 5 weeks and 24 after the MMI. Out of the 41 participants (mean age = 72.05 years; *SD* = 5.61, 27 male) originally assigned to the WCG, 33 completed the second survey after the waiting condition and 29 after the MMI. Furthermore, 10 participants wanted to attend a partner tandem, and 21 wanted to attend a group experience exchange online chat. Thirty-three participants stated that they used memory strategies during T1, 39 during T2, and 44 during T3.

### Procedure

The participants were randomly assigned to one of three groups before completing the pretests. We used stratified random sampling with gender as the relevant stratification variable. Participants received the testing links *via* email and completed the online surveys and tests (~60 min) from home on a computer or tablet. After that, they received all relevant study information and intervention content. They could independently divide the surveys into 2 units. If they chose to do so, then the online program could be continued from whatever test or question the participants last completed. In the following 5 weeks, all groups got weekly emails with procedure information and welcome and instruction videos. Thus, all groups received a comparable personal experience and instructional instrument.

The MMI group received weekly exercises, and background information promoting the six included areas *via* pdf-documents, emailed by the study investigator. Participants were encouraged to print the document, display it somewhere obvious in the house, and implement the exercises in their everyday life. The task units required only a small amount of time to complete, approximately 2–10 min each. Thus, the participants could easily integrate them into their daily routine. Participants could choose four of six preferred exercises and decide when and how intensively they would perform them to support their individual autonomy (Deci and Ryan, 2008). However, they were encouraged to do all exercises and activities either daily or at least 3–5 times per week, resulting in 60 min of activity per week. This amount of exercise is in line with general intervention intensity suggestions (e.g., review of lifestyle intervention studies for the elderly, Toman et al., 2018). Compliance with the instructions was moderated through a weekly questionnaire (~5 min) regarding their experience with and details on the task they practiced.

The CT group was assigned to a computer-based training program performed on a computer, tablet, or smartphone. Participants were asked to practice approximately 1 h per week, divided into 3 units of 20 min each. They were free to choose when to train. Regular training was meant to have been practiced at a challenging, but not overly-challenging, level. Compliance with the instructions was checked using the automatically transmitted data from the CT computer software and a weekly feedback questionnaire. Each of the participant’s progression history was retrieved weekly by the study investigator *via* the software program.

The WCG was informed that they were assigned to the waiting condition and would receive the MMI after T2. In the weekly emails, they received a link to fill in short weekly feedback surveys. The emails contained small talk about the weather, the season, and different jokes to keep communication at a similar level to the other groups. The participants could also send their favorite jokes to appear in the following email.

After the first 5 weeks of interventions at T2, all participants completed the between-tests. Then, the cognitive and the WCG received the same MMI information emails and the same documents with the MMI task and recommendation that the MMI group received during the first intervention time. The MMI group got five more MMI documents in the following 5 weeks, which each contained new exercises for the respective areas. Finally, all the participants accomplished the post-intervention tests and surveys 3–5 days after their last intervention week. If participants failed to complete the exercises more than three times despite receiving reminder emails from the study investigator, they were excluded from the study.

Participants who wanted to attend the supervised online exchange groups were randomly divided into groups of six participants. In these chats, we sent standardized weekly questions regarding, for example, their favorite task during the week or their task implementation experience, to encourage experience exchange. For the partner tandem, we introduced two participants by sending them the name and telephone number of the other person.

### Intervention Programs

In this study, we tested and compared two interventions. Both interventions were developed by the Department for Memory and Learning at the University of Bern (Switzerland). The CT tasks are part of an evaluated working memory training task collection designed as an application for use on tablets and smartphones (Studer-Luethi et al., 2016). The MMI implements findings from various applied studies that have tested cognitive, emotional, social, and motivational interventions, and which were shown to be beneficial for cognitive and affective health factors and against cognitive decline (WHO Guidelines, 2019). The MMI was developed by the Department of Memory and Learning at the University of Bern and evaluated previously in a cross-sectional study (Studer-Luethi et al., 2021).

#### Multimodal Intervention

The weekly task sheet (3–6 pages, see https://cdn1.site-media.eu/images/document/5816924/Study_Week3.pdf) contained exercise descriptions and links to short video demonstrations of various cognitive activation exercises to be performed daily or weekly. Furthermore, the task description contained psychoeducational information about each beneficial area written in highly accessible language. The beneficial areas and applied intervention tasks are the following: (1) *Physical health-related tasks*, with nutrition recommendations or physical exercises (e.g., drinking 2 L of water per day, drinking green tea, walking backward, or space- and body perception tasks); (2) *Coordination*, with physical coordination tasks (e.g., balance, juggling, fein- and basic motor tasks); (3) *Cognitive tasks*, with working-memory or attentive tasks and memory strategies (e.g., puzzles, classic memory tasks, and Loci- and Pegword-Method); (4) *Mindfulness*, with exercises for increased self-awareness (e.g., thankfulness-diary or breathing tasks); (5) *Creativity*, with generating ideas in new ways (e.g., painting, singing, or imagination tasks); and (6) *Community*, with ideas for social activities without physical contact (due to COVID-19) or communication skills (e.g., complimenting a friend *via* telephone or exchanging daily experiences); additional humor, with positive stimulus through jokes and cartoons.

Alongside the background information for each MMI task, each week concentrated on one cognitive health topic. This page included psychoeducational information about brain functions and neuroplasticity. The topics addressed all beneficial areas. Examples of topics are: “Music: massage for our brain,” “Use it or lose it,” and “Why our brain needs community.” Participants were encouraged to ask questions if they had any trouble understanding the provided information.

#### Cognitive Training

The CT used in this study is called “ihirn” (in English “ibrain,” see: https://ihirn.unibe.ch/app/tasks). The training tasks were completed on a computer, tablet, or smartphone. Generally, they consisted of the presentation of different stimuli (shapes, animals, balls, etc.), to which the user had to react with a button press according to the instructions. The training tasks were adaptive to the working memory capacity limits of the participant. The types of practice tasks are: (1) *n-back tasks*: Various *n*-back training tasks that require the continuous updating of information and are used to increase working memory performance; (2) *complex memory tasks*: Various tasks that require the processing and storage of information and the attention-ability to reacting to different stimuli (animals and Shrek family) depending on the instruction and the sequence, which must be memorized; and (3) *classic memory tasks*: This task requires information to be reproduced forward or backward, increasing memory span and training the working memory.

### Outcome Measures

The outcome measures that we predicted would be trained by the interventions were divided into three major categories: (1) Cognitive tasks through tests of classic memory, working memory, attention, and fluid intelligence; (2) transfer effects to reported activity in everyday life through reported variability and frequency of everyday life activities; and (3) additional transfer effects to reported personal variables in everyday life through questionnaires on well-being, stress, memory performance in everyday life, and attitude toward the aging brain.

#### Cognitive Measures

Cognitive measures were quantified for the following cognitive domains.

##### Classic Memory

We used a direct- and delayed recall-verbal memory test with an online-programmed adaptive version of the widely used neuropsychological tool for verbal memory span RAVLT (Rey Auditory Verbal Learning Test, Rey, 1941). The RAVLT was shown to be a valid and reliable psychometric instrument in neuropsychological assessment (Cronbach Alpha *α* = 0.80). Participants needed to remember 15 words that appeared one after another either directly or after a time delay (after 30 min).

##### Working Memory

We programmed an adapted version of the visual memory span test in combination with an n-back questionnaire, where participants needed to display the order of overlearned images (Della Sala et al., 1999; Jaeggi et al., 2003). The questionnaire asked to indicate when the presented word matched the stimulus that occurred in the series *n* steps before.

##### Attention

We measured reaction time and task switching flexibility in this task, which needs selective and sustained attention. Different pictures of objects were presented. Participants were requested to choose if the object was bigger or smaller than a football by pressing the corresponding button as fast as possible.

##### Fluid Intelligence

Participants performed an online-programmed version of the Raven’s Standard Progressive Matrices test (RPM; Raven, 2003) to test visual- and classification skills, spatial-constructive skills, and fluid intelligence (Cronbach Alpha *α* = 0.87, convergent validity *r* = 0.67). The participants saw a 3 × 3 matrix of shapes presented with the last shape missing and were required to choose which of six to eight items completed the pattern. Participants were given 10 min to complete the task. The number of correctly answered items served as the dependent variable

#### Self-Reported Measures of Activity in Everyday Life

Transfer effects to activity in everyday life were quantified using self-reported measures in the following domains:

##### Variability of Everyday Life Activities

We assessed the individual activity variability in everyday life with the questions, “What do you do to keep yourself mentally fit and healthy? And how often to you participate in these activities?.” The answer options included 12 activities, such as memory exercises (e.g., puzzles, sudoku, and crossword puzzles), sports, musical activities, dancing, taking care of grandchildren, gardening, and online memory programs (e.g., CogniFit and NeuroNation), or social engagement (e.g., activities in clubs) and one option designated as “other activity, namely…,” where participants could add one or more activities that they used to support their mental health. Each of the 13 answers options was accompanied by a Likert scale ranging from 1 “never” to 5 “daily/very often.” Then, we calculated the variability score by adding the number of activities and multiplying this score with how often they professed to participate in the respective activity.

##### Frequency of Everyday Life Activities

We assessed the frequency with which participants engaged with activity with this prompt: “Please estimate the daily average amount of time (in minutes) you spend engaging with activity (e.g., gardening, walking, cooking, and coordination/or balance exercises).”

#### Self-Reported Personal Variables in Everyday Life

Transfer effects to cognitive and affective perception factors were quantified with self-reported measures for the following domains.

##### Well-Being

We used the German version of the Positive and Negative Affect Schedule (PANAS, Watson et al., 1988), which consists of six positively-coded word items (e.g., “thankful,” “excited,” and “determined”) combined with six negatively-coded word items (e.g., “afraid,” “worried,” and “angry”). The PANAS demonstrated good validity and reliability (Cronbach Alpha *α* = 0.86).

##### Perceived Stress

The feeling of being stressed in everyday life was measured with the short version of the perceived stress questionnaire, which produced good psychometric properties (Cronbach Alpha *α* = 0.80–0.86; Fliege et al., 2009). Answers to the 11 items (e.g., “I feel well-rested,” “I have a lot of worries,” or “I am full of energy”) were given on a Likert scale ranging from 1 (strong disagreement) to 4 (strong agreement).

##### Memory in Everyday Life

We used the Prospective and Retrospective Memory Questionnaire (PRMQ; Smith et al., 2010) as a self-report measure for memory in everyday life, which also showed acceptable validity and reliability (Cronbach Alpha *α* = 0.74–0.86). The questionnaire consists of 16 questions, eight asking about retrospective memory failures (e.g., “Do you forget what you watched on TV the previous day?”) and eight concerning prospective failures (e.g., “Do you decide to do something in a few minutes time and then forget to do it?”). Answers were given on a Likert scale ranging from 1 (never) to 5 (very often).

##### Attitude Toward the Aging Brain

We evaluated the participant’s attitude with the question: “On a scale from 0 to 10, how do you rate your attitude toward the aging brain?,” with 0 meaning “I am afraid of mental deterioration (e.g., reduced performance and speed)” and 10 meaning “I am confident that the brain can continue to learn and perform well.”

#### Additional Measures

##### Memory Strategy Use

We measured the use of memory strategies during the three cognitive tests by asking at the end of the test if they used memory strategies during the test tasks.

##### Social Exchange

We measured the influence of the optional attendance in online exchange groups or partner tandem on the depended variables.

### Data Analysis

The linear mixed model was performed with the software R (Dalgaard, 2010). In the analysis, we modeled the values of the factors of interest between the three independent study groups over three time points. The dependent variables were interval-scaled, and the assumption of a normal distribution was checked with the Kolmogorov–Smirnov test in IBM SPSS Statistics 26.0 Software (IBM Corp, 2020).

Due to missing entries in the data, multiple imputation by chained equations (MICE) was applied as an imputation method in all analyses (consisting of 30 imputation steps). Outlier removal was performed automatically for each target variable for each group at each time point. Data points were classified as outliers if they were located further from the median than four times the median absolute deviation, an approach considered as a very conservative outlier detection measure (Leys et al., 2013).

Since the mixed model was fitted with interaction terms between time point and group, each group’s individual performance change was modeled. For each of the target variables, we fitted a linear mixed model with patient ID as a random intercept (fixed effects of the linear mixed model in the notation of the used nlme R-package: target variable ~ timepoint * group + gender + age + use of strategies + partner tandem + exchange group).

## Results

We examined the pretest data to evaluate possible differences between the three groups. There were no group differences in gender, age, years of education, civil status, or self-rated health condition.

### Intervention Commitment

#### Multimodal Intervention

Around 78.85% of participants attending the MMI (during the intervention time T1–T2 and T2–T3) responded in the weekly questionnaires on average that they “have done all the exercises regularly,” 14.42% responded that they “have mostly done the exercises,” and 6.73% (16 people) “have not done the exercises” and therefore needed to be excluded. About 17.69% stated that they did not watch the linked videos. About 57.93% indicated that they trained 5–10 min every day, 24.39% indicated 10–20 min, 7.32% more than 20–30 min, and 10.37% more than 30 min. In summary, 22.18% indicated that they found the exercises to be very interesting and educational, 56.04% pretty educational, 17.23% a bit, and 4.55% not at all.

#### Cognitive Training

In the cognitive training group, seven (21%) of 33 people who attended the whole training but did not reach the predetermined training time were excluded. About 27.66% indicated that they trained 5–10 min every third day, 42.55% 10–20 min, and 23.40% more than 30 min. About 12% stated that they liked the exercises very much, 12% liked them, 16% a bit, and 4.3% not very much.

### Cognitive Measures

#### Classic Memory (Direct Recall)

There was a significant improvement in the word span direct recall test for the CT (*β* = 2.17, *p* = 0.03) group and the WCG (*β* = 1.73, *p* = 0.04) from T1 to T2 and a positive tendency for the MMI group (*β* = 1.30, *p* = 0.12). During the intervention time with the MMI from T2 to T3, the groups showed no further significant improvements to their performances. The biggest benefits were found for the WCG (*β* = 1.80, *p* = 0.05). There were no significant time × group interactions. Additionally, participants who used memory strategies during the test performed significantly worse (*β* = −1.40, *p* = 0.02) than those who did not, and there was no significant effect for group exchange or partner tandem.

#### Classic Memory (Delayed Recall)

**Figure 2:**
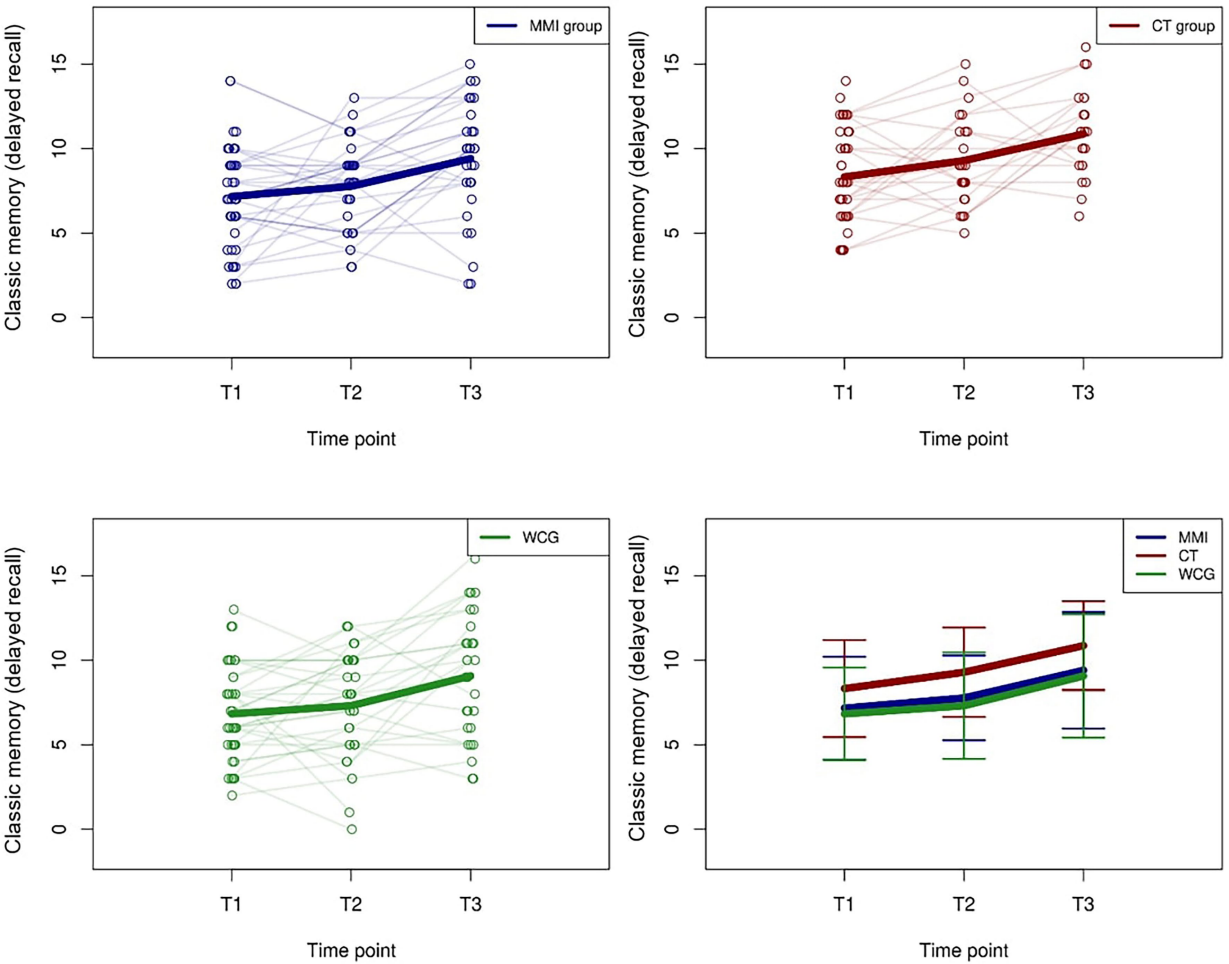
Trajectories of “classic memory (delayed recall)” values for the MMI group, the cognitive training (CT) group, and the WCG. The thick lines indicate the mean values.

There was a significant positive effect in the word span delayed recall test (after 30 min) for the MMI group (*β* = 1.35, *p* = 0.03), and the WCG (*β* = 1.39, *p* = 0.04) and a positive tendency for the CT (*β* = 1.61, *p* = 0.07), during their intervention phase from T2 to T3, where all groups received the MMI (Figure 2). There were no significant effects during T1–T2 and no significant time × group interactions. Additionally, there was again a significant negative difference for participants who used strategies during the test (*β* = −1.00, *p* = 0.01) compared to those who did not, and no significant effect for group exchange or partner tandem.

#### Working Memory

There was a significant decline in the CT group (*β* = −1.64, *p* < 0.01) from T1 and T2 and a tendency to improve in the MMI group (*β* = 0.60, *p* = 0.07). The MMI group performance was significantly better than the CT group (*β* = −2.24, *p* < 0.01) and the WCG (*β* = −0.96, *p* = 0.04) from T1 to T2. There was a significant improvement from T2 to T3 in the CT group (*β* = 1.95, *p* < 0.01) and a significant difference between the MMI group and the CT group (*β* = 1.95, *p* = 0.01) in favor of the CT group. Additionally, there was no significant effect of the use of memory strategies and no significant effect for group exchange or partner tandem.

#### Attention

**Figure 3:**
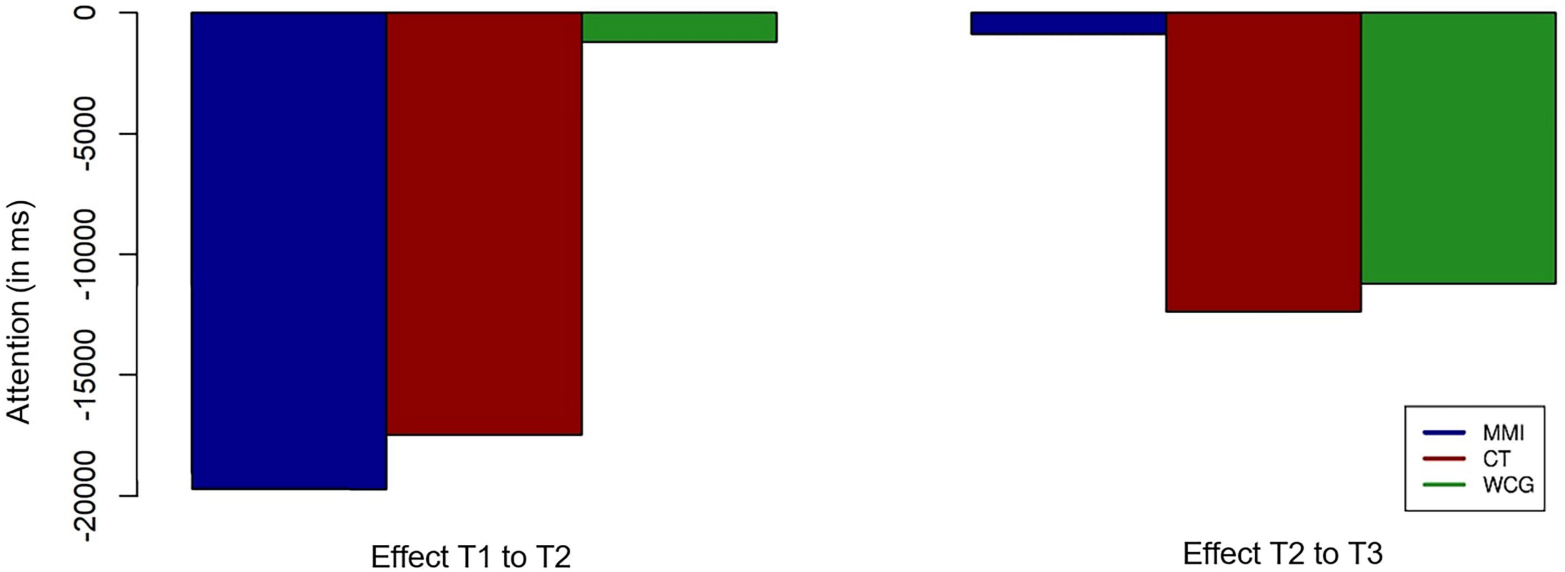
Visualization of the time effects in the “Attention” variable in milliseconds for the MMI group, the CT group, and the WCG. Stars indicate the significance of effects for individual groups and group comparisons.

There were no significant effects of any group from T1 to T2 and from T2 to T3. However, a visualization of the presented effects in Figure 3 shows that reaction times tended to decrease.

#### Fluid Intelligence

There was a significant positive effect in the Raven’s Progressive Matrices test for the MMI group (*β* = 1.81, *p* = 0.03) and WCG (*β* = 1.73, *p* = 0.03) during the intervention time from T1 to T2. There were no significant effects from T2 to T3 and no significant time × group interactions. Additionally, there was no significant difference between people who used memory strategies and those who did not, with no significant effect for group exchange or partner tandem.

### Self-Reported Personal Variables in Everyday Life

#### Well-Being

There was a significant effect of positively-affected well-being perception between T1 and T2 for the MMI group only (*β* = 1.00, *p* = 0.01). There was also a significant positive effect during T2–T3 for the CT group, which then received the MMI (*β* = 1.24, *p* = 0.01). However, there were no significant time × group interactions. Additionally, there was no significant effect for group exchange or partner tandem.

#### Stress

There was a significant effect of feeling less stressed for all groups between T1 and T2 (MMI group: *β* = −0.81, *p* < 0.01; CT group: *β* = −0.40, *p* = 0.04; and WCG: *β* = −0.52, *p* < 0.01). And a significant difference between the MMI group and the CT in favor of the MMI (*β* = 0.40, *p* = 0.02). However, there was an increase of feeling stressed between T2 and T3 in all groups (MMI group: *β* = 0.73, *p* < 0.01; CT group: *β* = 0.49, *p* = 0.03; and WCG: *β* = 0.50, *p* = 0.04). There was no significant effect for group exchange or partner tandem.

#### Memory Performance in Everyday Life

There was a significant time × group interaction between the MMI group and WCG during T2–T3 (*β* = −1.72, *p* = 0.04), for the prospective memory, in favor of the MMI group, but no other significant increase between the time points. However, all groups showed a slightly positive influence during T1 to T2 (MMI group: *β* = 0.66, *p* = 0.24; CT group: *β* = 0.45, *p* = 0.49; and WCG: *β* = 0.31, *p* = 0.60). Additionally, there was a significant negative relation between “age” and this target variable (*β* = 0.14, *p* = 0.01).

#### Attitude Toward the Aging Brain

**Figure 4:**
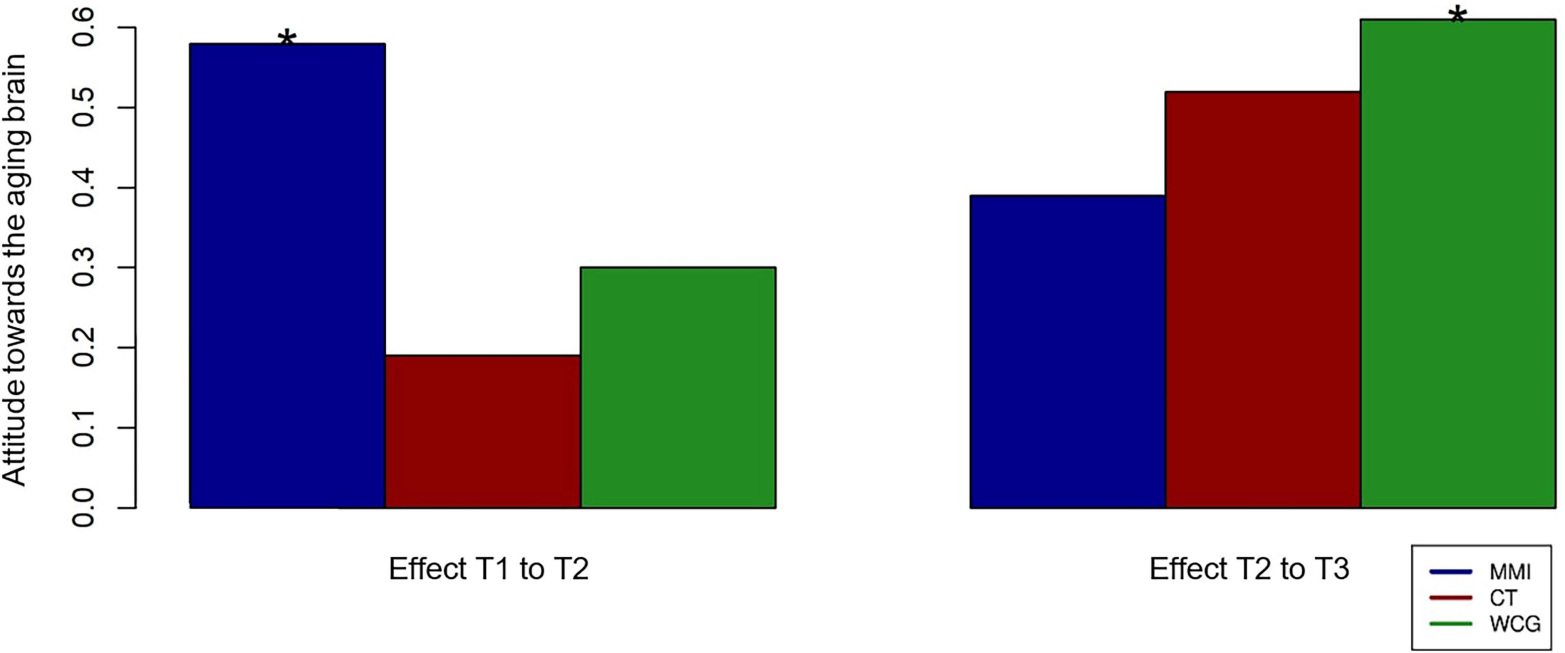
Visualization of the time effects in the “Attitude towards the aging brain” variable for the MMI group, the cognitive training (CT) group, and the WCG. Stars indicate the significance of effects for individual groups and group comparisons.

There was a significant positive effect of attitude change between T1 and T2 for the MMI group (*β* = 0.58, *p* = 0.01). There was also a significant positive effect during T2 to T3 for the WCG (Figure 4), which then received the MMI (*β* = 0.61, *p* = 0.01). However, there were no significant time × group interactions. Additionally, there was no significant effect for group exchange or partner tandem.

### Self-Reported Measures of Activity in Everyday Life

#### Variability of Everyday Life Activities

**Figure 5:**
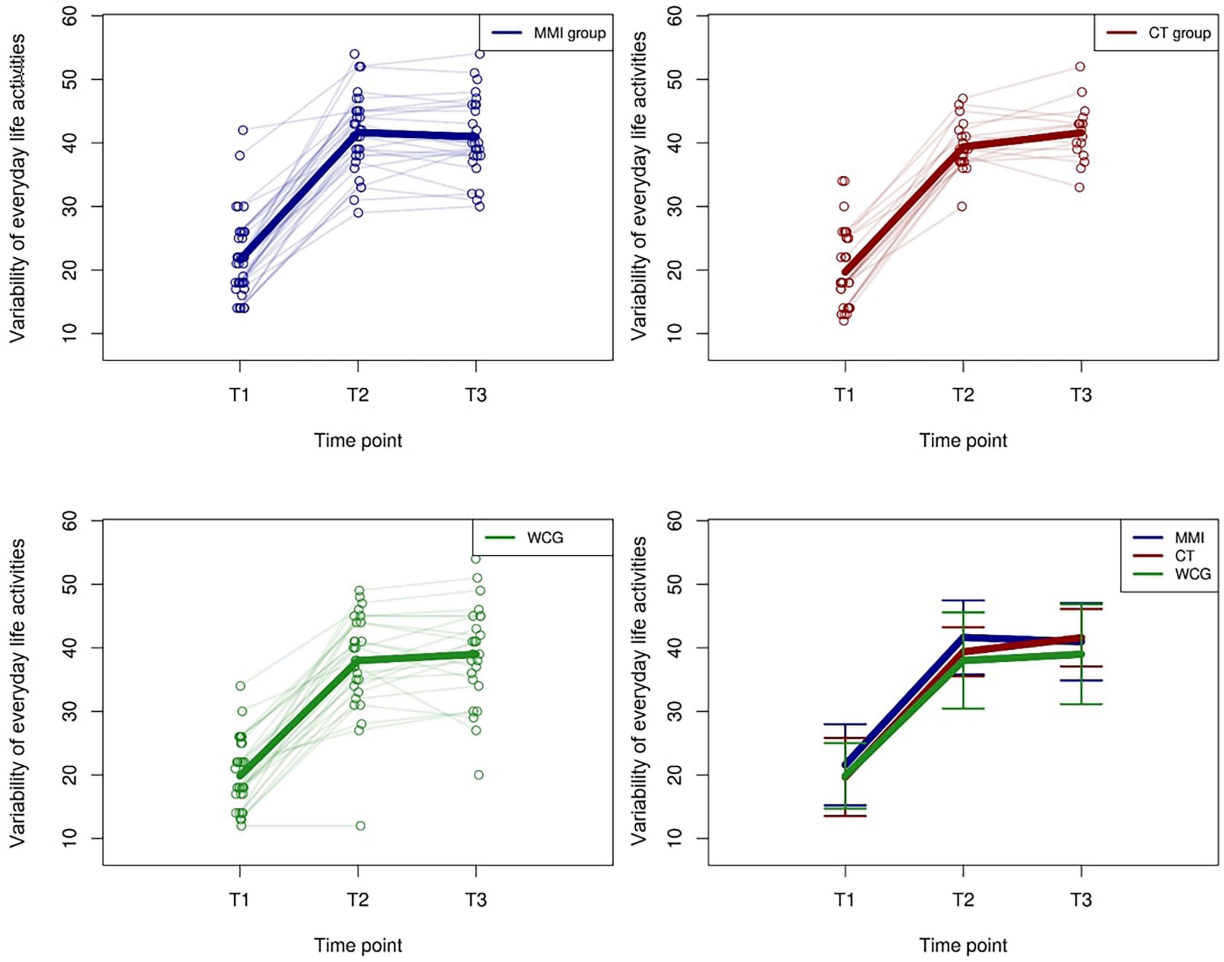
Trajectories of “Variability of everyday life activities” values for the MMI group, the CT group, and the WCG. The thick lines indicate the mean values.

There was a significant positive effect for all groups between T1 and T2 (MMI group: *β* = 19.41, *p* < 0.01; CT group: *β* = 19.31, *p* < 0.01; and WCG: *β* = 18.19, *p* < 0.01). There was no significant difference for any group between T2 and T3 (Figure 5). Additionally, women gained significantly more variability in their everyday life activities than men (*β* = 2.61, *p* = 0.02). There was no significant difference between people who used memory strategies during the test and those who did not, with no significant effect for group exchange or partner tandem.

#### Frequency of Everyday Life Activities

There was a significant improvement for all groups between T1 and T2 (MMI group: *β* = 324.38, *p* < 0.01; CT group: *β* = 164.99 *p* < 0.01; and WCG: *β* = 202.80, *p* < 0.01). There was a significant decrease in the frequency for the MMI group (*β* = −113.19, *p* = 0.04) between T2 and T3 and no significant difference for the other group. There was no significant difference between people who used memory strategies and those who did not, with no significant effect for group exchange or partner tandem.

## Discussion

This single-blind controlled longitudinal study investigated the effects of MMI and CT, compared to a WCG, on cognitive and affective factors, everyday life memory performance, and daily activity in elderly people. The MMI was autonomy-supportive and combined six intervention areas that have been shown to be particularly beneficial for cognitive and affective health; namely physical, cognitive, social, mindful, coordinative, and creative activation, combined with psychoeducational information. We assessed cognitive performance and self-reported variables at three different time points (T1 and T2 after 5 weeks, T3 after 10 weeks). From T2 to T3, all groups accomplished the MMI.

Our results showed general improvements through all groups on most cognitive and affective factors and an increase in the number and variability of activities in everyday life after the intervention phase. A significant group difference was only found for working memory performance in favor of the MMI compared to the CT and the WCG. *Post hoc* analysis on cognitive performance of MMI to CT indicate similar (classic memory and attention) or better (working memory) effects. Results on further transfer variables, such as attitude toward the aging brain and well-being, indicate some trends for differential effects in favor of the MMI group. Also, the MMI group reported the highest perceived improvements in all self-reported personal variables in everyday life (e.g., memory performance in everyday life and stress). Lastly, the findings suggest that scientific information about multimodal activity encourages participants to be more cognitively and variably active in their everyday lives.

### Effects on Cognitive Performance

We predicted that when people over 65 years take part in an MMI or CT, there would be measurable increases in their performances on classic memory, working memory, attention, and fluid intelligence tasks. Indeed, the outcomes demonstrate a trend for comparable performance increases after the MMI and CT. Also, we hypothesized that CT has a bigger influence on task-specific outcomes than on further transfer factors, such as fluid intelligence. The results showed inconsistent findings regarding this hypothesis. In the following section, the results are discussed in more detail.

The “classic memory (direct recall)” task was a near task-specific measure, which was trained in the CT with classic memory tasks. Our *post hoc* analysis indicated a positive near task-specific training effect of the CT group compared to the MMI, though also the WCG improved their performance. The effects demonstrate a positive trend for CT, which is in line with most research (Ball et al., 2002; Jaeggi et al., 2011), though there were no group interactions. The MMI had a higher impact on the “classic memory (delayed recall)” task. From T2 to T3, while all groups received the MMI, all groups improved their performance, although the MMI did not include exercises like the “classic memory” task used in the CT (*cf.* Hong et al., 2021 for a multi-domain cognitive training). We assume that the MMI, which included cognitive exercises too, supports the storage and updating of information (*cf.* Ngandu et al., 2015).

Furthermore, the performance on both classic memory tasks was negatively related to the use of memory strategies. We assume that this effect could be related to the speed of the task (each word was only presented for 2 s), which allowed very little time to mentally link pictures or stories. Previous research found inclusive results on the effects of memory strategies on memory. Some showed no transfer effect to untrained memory tasks (Saczynski et al., 2010), and some showed positive effects on performance of other cognitive tasks (Gamino et al., 2010; Gross et al., 2012; Li et al., 2016, for a meta-analysis). However, the negative effect of strategy use on memory performance seems surprising.

Regarding “working memory” performance, the MMI group showed a significantly higher increase than the CT group and the WCG. *Post hoc* analysis revealed a significant decline in test performance in the CT group between T1 and T2. This result is difficult to interpret, especially since the n-back task was one of the training tasks in the CT. Previous research mostly showed positive effects of CT on n-back related tasks (Jaeggi et al., 2010; Studer-Luethi and Meier, 2020). However, after the CT group received the MMI, they significantly improved their performance compared to the MMI group. A relatively similar result was found by Burgener et al. (2008), where a cognitive intervention group received attention-educational training and showed decreased cognitive outcomes after 20 and 40 weeks, compared to a MMI group (Taiji exercises, cognitive-behavioral therapies, and support group) which reached positive outcomes for cognitive tasks.

Regarding attention performance, there is a trend for improved performance for the MMI group and the CT group during the first intervention phase (T1-T2). However, there were no significant group or interaction effects. From T2 to T3, when all groups received the MMI, the WCG could also improve their performance, whereas the MMI group showed no further change in their performance. This finding is in line with different studies showing the positive influence of multidomain interventions with included cognitive exercises or single domain cognitive interventions on attention performance (e.g., Oswald et al., 2006; Eggenberger et al., 2015; Gajewski et al., 2020).

Regarding “fluid intelligence” performance, *post hoc* analysis suggests that the MMI but not the CT group profited from the intervention, but also that the WCG significantly increased their test performance. One possible reason for this performance gain could be the increased activity and variability of activities which might have stimulated intellectual behavior (Sabbagh et al., 2022). Alternatively, motivation or a simple re-test effect is possible explanations.

Our results are in line with findings from other MMI or multidomain interventions, which mainly tested two to three combined interventions (mostly cognitive training, physical activity, and/or diet, *cf.* Ngandu et al., 2015), that showed effects on some cognitive performances, such as working memory, episodic memory capacity, or attention (*cf.* Kane et al., 2017, for review) or fluid intelligence (Zwilling et al., 2020). Regarding the lack of improvement after CT, our findings align with the current research for CT (Sala et al., 2019 for a second-order meta-analysis on cognitive training), which concludes that several CT studies did not find specific effects on working memory or transfer effects on fluid intelligence.

### Effects on Frequency and Variability of Everyday Life Activities

We predicted transfer effects for the MMI group on the frequency and variability of everyday life activities. Indeed, our results demonstrate a significant effect of the MMI on the frequency and variability of everyday life activities. However, this effect was also observed in the CT and WCG groups after the first intervention phase. One reason for this result could be that participants were not blind to the aims of this study. That is, all participants were informed through the recruitment flyers about the predicted benefits of comprehensive MMI activities. This beneficial effect that we found across all groups aligns with the health belief model theory (Schwarzer, 2004). It states that a factor’s perceived susceptibility and severity as well as the perceived benefits and barriers of a related behavior influence people’s health behavior. For example, older participants feel more susceptible to more severe cognitive decline than younger participant groups (Bowen et al., 2019). The study goal information probably led participants to guess as to the beneficial preventative impact such comprehensive MMI activities might have against cognitive decline. Furthermore, the study description might have particularly interested people motivated to change their behavior (*cf.*, Lam, 2015). Our participants’ high motivation was evidenced by several positive feedback emails and notes that the WCG already sent during their waiting time. However, there was no further significant positive effect of “activity frequency” or “variability” from T2 to T3, but participants maintained their claimed activity and variability level (MMI group slightly decreased their level in relation to the reached gain). This finding possibly shows a ceiling effect for activity change (Austin et al., 2002). The hypothesis that MMI has further transfer effects on activity outcomes is partially confirmed in that in particular, knowledge of the preventive effects of multimodal activities seemed to have an important influence.

### Effects on Self-Reported Personal Variables in Everyday Life

We predicted far transfer effects on memory performance in everyday life, well-being, stress, and attitude toward brain health for the MMI group compared to the CT group. The results showed no group differences in any self-reported personal variables in everyday life. Though, in *post hoc* analysis, the effects indicate a trend that supports the assumption that MMI leads to more transfer effects compared to the CT and the WCG. That is, the MMI group showed the highest improvements in all self-reported variables in everyday life (memory performance in everyday life, stress, well-being, and attitude toward the aging brain). In particular, well-being and attitude toward the aging brain were only significantly influenced by the MMI group compared to the other groups in T1 and T2. Regarding the phase from T2 to T3, where all groups received the MMI, the other groups also reported improvements for each of the different self-reported variables in everyday life (some were significant, e.g., attitude toward brain health for the WCG and well-being in the CT group), which could indicate some of the beneficial effects of the MMI. Our results did not find far transfer effects for the CT, except for variables stress, which was significantly lower in all groups and memory in everyday life, which increased slightly in all groups. Our findings align with other research, where CT produced no further effects on transfer variables such as episodic memory, everyday life performances, or quality of life (*cf.* Gates et al., 2019; Hudes et al., 2019; Roesch-Ely and Baum, 2019). In comparison, the positive influence of multidomain interventions on mood was also found in other studies, including physical and creative exercises with older people in nursing homes (*cf.* Hons et al., 2016, for a review).

We assume that the positive influence on a positive attitude toward brain health in the MMI group is primarily due to the psychoeducational information they were given on neuroplasticity (e.g., Kays et al., 2012). This positive attitude change may result in other positive effects. That is, studies researching negative and positive age stereotypes demonstrated a connection between a positive attitude and a slower and smaller amount of cognitive decline (Levy et al., 2016). Dolcos et al. (2018) even promotes the possibility of attitude-changing neuroplasticity mechanisms and a subsequently positive influence on health and well-being.

It is possible that the results were also influenced by some other differences between the interventions. Compared to the specific CT intervention, the MMI approach held more variability and was more easily integrable in everyday life. Furthermore, most of the exercises were shorter (2–5 min in the MMI vs. a minimum of 15 min in the CT) and allowed combination with other daily habits (e.g., coordination task while cooking or walking). Lastly, there was less need for an effective digital tool or technological know-how in the MMI. However, the questionnaire on perceived stress and memory in everyday life indicated some change in the CT group toward lower stress and higher memory performance. These trends are consistent with intervention studies over 10 years, which showed significant benefits of CT in everyday memory perception (Rebok et al., 2014), and less stress-related perception in CT groups (Gavelin et al., 2015). Also, compared to other studies, we used a shorter intervention time of 5 weeks instead of eight or more weeks (Gajewski and Falkenstein, 2012; Küper et al., 2017). Therefore, there might have been further beneficial effects on memory in everyday life and stress in the CT group should we have chosen a longer intervention time.

### Additional Intervention Effects

Across all groups, we found comparable performance improvements and self-reported measures of personal variables in everyday life, although the strength of the effect differed between the experimental groups. We assume that this difference could be driven by the large amount of activity variability and frequency change in all groups during T1 and T2. Therefore, our findings also support the potential of informing people about the effectiveness of multimodal activities on motivating them to change their everyday activities (*cf.* Jensen et al., 2010; Avitabile and De Hoyos, 2018; Bergman and Chan, 2021; Maldonado and De Witte, 2021).

We found no effects for the group exchange chat or partner tandem. However, this finding is based on a small subsample of the participants who chose to engage in the exchange groups, and the exchange was not intensive overall. Some groups even stopped after a few weeks. Based on previous findings (Holt-Lunstad et al., 2010; Harling et al., 2020; Van Lange and Columbus, 2021), we hypothesize that such tandem or group exchange possibilities could lead to significant influences under different circumstances.

### Limitations of the Study

The study had several limitations. First, despite enlarging our recruiting phase and motivating more than 130 participants to attend, we did not reach our goal of 240 participants for a stronger power and effect size. Second, we chose to do an online experiment with an online survey and tests. This is not a limitation *per se*, but several points need to be considered. Online experiments have low control over the environment (distractions, such as lighting, noise, etc.), and the reliability of the tests is influenced by user systems (internet connection speed), measurement precision (sliders and reaction time), and/or the monitor size of the user’s computer. The validity of the experiment is influenced by bots, participant motivation, fraud (“defective” or other person responding), and task comprehension. Further, 68% of the included participants presented a high education level, which might implicate a limitation in the validity of our results with regard to the wider population. Lastly, we did not differentiate between multiple processes that could have played a role (such as neurobiological and psychological mechanisms) but tested the overall effect of the MMI. Therefore, our results do not indicate which activated mechanisms of the MMI are more beneficial than others.

### Conclusion

So far, evidence for the effectiveness of MMI is inconclusive, though trends indicate that it could be beneficial to combine interventions to prevent cognitive decline (*cf.* Kane et al., 2017 for a review). Our results for a MMI that combined six intervention areas, compared to a specific computer-based CT and a WCG, demonstrate promising results including an increase in the number and variability of activities in everyday life after all interventions. These findings suggest that information about multimodal activities promotes/motivates participants to be more cognitively and variably active in their everyday life. It is encouraging to see older people being so motivated to adapt their lifestyle through exposure to scientific information. We found that the MMI group significantly improved working memory performance in comparison to the CT group and the WCG. Also, the effects on self-reported variables showed interesting trends in favor of the MMI, such as increased well-being and attitude toward the aging brain as well as improved memory in everyday life. These trends show the potential of MMIs, which combine different beneficial areas with psychoeducational information. Compared to CT, similar (classic memory and attention) effects on cognitive variables were also found through the MMI, though there was only one time × group interaction. However, both approaches seem to address and positively influence cognitive factors.

Compliance and Commitment answers suggest a more positive reaction to MMI activities compared to CT. We assume the participants, to some extent, prefer MMI exercises because of their ability to be combined easily with other daily activities as well as the autonomy-supportive and psychoeducational approach (Deci and Ryan, 2008; Clare et al., 2015). Based on these assumptions, we speculate that further long-term effects of the MMI could be found on lifestyle change and the increase in cognitive and affective health factors (*cf.* Knittle et al., 2018 for a review).

We recommend that further research should not focus especially on specific interventions, such as CT, but on positively influencing changes to everyday activities to promote the cognitive and affective health of older people. This aligns with other research promoting an active lifestyle instead of specific training for older adults (e.g., Küster, 2018), and combinations of multimodal activities to minimize cognitive decline, maintain activity, and positively influence attitudes and well-being (Burgener et al., 2008; Ngandu et al., 2015). We further suggest implementing multimodal activities that are easily combined and added to everyday life activities (*cf.* Clare et al., 2015). Further studies should measure the beneficial effect of cognitive and affective activity on its stability on cognitive and affective health factors over time. Also, further research should examine the wider and especially differential effects of MMIs. In line with other studies (Lorenza, 2020), we suggest that CT programs should be studied for individual differences. When intervention tasks are personalized to participants’ lifestyles, we hypothesize that we could see further-reaching and stronger transfer effects. Therefore, we recommend further research in standardized, personalized interventions. Also, MMI should be tested and implemented earlier in life than at 65 years of age. In summary, we promote the implementation of scientific information on preventive factors against cognitive decline (e.g., through MMI), more so than a focus on CT alone, as an approach for positively influencing everyday life activities and for producing positive near (cognitive performances) and further-reaching transfer effects (well-being and attitude).

## Data Availability Statement

The raw data supporting the conclusions of this article will be made available by the authors, without undue reservation.

## Ethics Statement

The studies involving human participants were reviewed and approved by the Ethics Committee of the University of Bern has approved the study. The Code is 2021-09-00001. The patients/participants provided their written informed consent to participate in this study.

## Author Contributions

MB: recruiting, data collection, intervention preparation, data analysis, and writing of the manuscript. AS and SF: contributed to the study development and manuscript. RS: recruiting and contributed to the study development. GR: data preparation. MH: contributed to the study administration. BS-L: intervention development and preparation, contributed to the study development, and writing of the manuscript. All authors contributed to the article and approved the submitted version.

## Conflict of Interest

The authors declare that the research was conducted in the absence of any commercial or financial relationships that could be construed as a potential conflict of interest.

## Publisher’s Note

All claims expressed in this article are solely those of the authors and do not necessarily represent those of their affiliated organizations, or those of the publisher, the editors and the reviewers. Any product that may be evaluated in this article, or claim that may be made by its manufacturer, is not guaranteed or endorsed by the publisher.

## Abbreviations

COVID-19: Coronavirus disease of 2019
MMI: Multimodal training
CT: Cognitive training
WCG: Waiting control group
