# Supplementary material for: A Randomized Controlled Trial Study of a Multimodal Intervention vs. Cognitive Training to Foster Cognitive and Affective Health in Older Adults
Source: Front Psychol. 2022 Jun 20;13:866613. doi: 10.3389/fpsyg.2022.866613 (PMC9251428; doi:10.3389/fpsyg.2022.866613)
Supplement: Supplementary file 1 [file Data_Sheet_1.PDF]

A randomized controlled trial study of a multimodal intervention vs. cognitive training to foster cognitive and affective health in older adults

Maria Brasser, Sascha Frühholz, Andres R. Schneeberger, Gian G. Ruschetti, Rahel Schaerli, Michèle Häner, Barbara Studer-Luethi

Data Analysis Code for R

```
est <- as.numeric(attributes(res)$value)

colnames(res_tbl) <- c('Estimate', attributes(res)$names)

res_tbl[c("Gruppe_1"),] <- c(est, res$F, res$`num df`, res$`den df`, res$`Pr(>F)`)

### Second group:

imp_fit <- lapply(comp.out_l, function(x){

dd <- x

dd$Gruppe <- factor(dd$Gruppe, levels = c(2, 1, 3))

dd$Timepoint <- factor(dd$Timepoint, levels = levels(x$Timepoint)[c(2,1,3)])

lme(fixed = frml, data = dd, random = ~1|ID)

})

### Linear hypothesis:

res <- linearHypothesis(as.mira(imp_fit), "-1*TimepointT1_ - 1*TimepointT3_ = 0")

est <- as.numeric(attributes(res)$value)

res_tbl[c("Gruppe_2"),] <- c(est, res$F, res$`num df`, res$`den df`, res$`Pr(>F)`)

### Third group:

imp_fit <- lapply(comp.out_l, function(x){

dd <- x

dd$Gruppe <- factor(dd$Gruppe, levels = c(3, 1, 2))

dd$Timepoint <- factor(dd$Timepoint, levels = levels(x$Timepoint)[c(2,1,3)])

lme(fixed = frml, data = dd, random = ~1|ID)

})

### Linear hypothesis:

res <- linearHypothesis(as.mira(imp_fit), "-1*TimepointT1_ - 1*TimepointT3_ = 0")

est <- as.numeric(attributes(res)$value)

res_tbl[c("Gruppe_3"),] <- c(est, res$F, res$`num df`, res$`den df`, res$`Pr(>F)`)

### Add significance:

res_tbl <- as.data.frame(round(res_tbl, digits = 2))
```

A randomized controlled trial study of a multimodal intervention vs. cognitive training to foster cognitive and affective health in older adults

Maria Brasser, Sascha Frühholz, Andres R. Schneeberger, Gian G. Ruschetti, Rahel Schaerli, Michèle Häner, Barbara Studer-Luethi

```
res_tbl$sig <- ifelse(res_tbl$`Pr(>F)`<apha, yes = '*', no = '')

### Extract object:

t1t2vst2t3_res <- res_tbl

#****************************************************************************

#   PLOT EFFECTS   ####

#****************************************************************************

### Generate needed table format for grouped barplot:

effpl <- matrix(NA, nrow=3, ncol=4)

rownames(effpl) <- rownames(t1t2_res)[1:3]

colnames(effpl) <- c('T1T2_Effect', 'T2T3_Effect', 'T1T2_sig', 'T2T3_sig')

### Fill up table:

effpl[,'T1T2_Effect'] <- t1t2_res[1:3, 'estimate']

effpl[,'T2T3_Effect'] <- t2t3_res[1:3, 'estimate']

effpl[,c("T1T2_sig")] <- t1t2_res$p.value[1:3]<apha

effpl[,c("T2T3_sig")] <- t2t3_res$p.value[1:3]<apha

if(creatPlots){

### Start device:

png(filename = paste0('./Figures/', y, '_effectPlot.png'), width = 20,

height = 10, pointsize = 10, units = 'cm', res = 300)

### Generate basic barplot:

yrang <- range(effpl[,1:2])  # Raw y range

toadd0 <- dist(yrang)/10

toadd <- sign(yrang)*toadd0

yll <- yrang+toadd   # ylims

### Take minimally zero as lower ylim:

yll[1] <- min(c(0, yll[1]))

yll[2] <- max(c(0, yll[2]))

bb <- barplot(effpl[,1:2], beside = TRUE, ylim = yll, col=cls)

### Add legend:

legend('bottomleft', legend = rownames(effpl), col = cls, pch=15)
```

A randomized controlled trial study of a multimodal intervention vs. cognitive training to foster cognitive and affective health in older adults

Maria Brasser, Sascha Frühholz, Andres R. Schneeberger, Gian G. Ruschetti, Rahel Schaerli, Michèle Häner, Barbara Studer-Luethi

```
### Add significance stars:

### Group significances:

displ <- abs(toadd/5)[1]

if(sum(effpl[,3:4])>0){   # Only in case there are sig results

### Draw stars:

text(x = bb[effpl[,3:4]==1],

y=(effpl[,1:2][effpl[,3:4]==1]) + sign((effpl[,1:2][effpl[,3:4]==1]))*displ, label='*', cex=2)

}

### Add comparison stars:

tckl <- abs(toadd/10)[1]   # Good ticklength

cmpCd <- combn(1:3, m = 2)   # All comparisons

yy <- max(yll) - 5*tckl   # First height to plot lines

### T1-T2 comparisons:

t1t2C_sig <- t1t2_res[4:6,'p.value']<apha

for(i in 1:length(t1t2C_sig)){   # Iterate through the groups

if(t1t2C_sig[i]){

xx <- bb[,1][cmpCd[,i]]

### Plot lines:

lines(xx, rep(yy, 2))

### Add star:

text(mean(xx), y = yy + tckl/2, labels = '*', cex=2)

### Update height:

yy <- yy + tckl

}

}

### T2-T3 comparisons:

t2t3C_sig <- t2t3_res[4:6,'p.value']<apha

for(i in 1:length(t2t3C_sig)){   # Iterate through the groups

if(t2t3C_sig[i]){

xx <- bb[,2][cmpCd[,i]]
```

A randomized controlled trial study of a multimodal intervention vs. cognitive training to foster cognitive and affective health in older adults

Maria Brasser, Sascha Frühholz, Andres R. Schneeberger, Gian G. Ruschetti, Rahel Schaerli, Michèle Häner, Barbara Studer-Luethi

```
### Plot lines:

lines(xx, rep(yy, 2))

### Add star:

text(mean(xx), y = yy + tckl/2, labels = '*', cex=2)

### Update height:

yy <- yy + tckl

}

}

dev.off()

}

#******************************

### Final return object:

L <- list("mssTbl_fin"=mssTbl_fin, "md_res"=md_res,

"t1t2_res"=t1t2_res, "t2t3_res"=t2t3_res,

"t1t2vst2t3_res"=t1t2vst2t3_res, "y"=y, "effpl"=effpl, cols=cls)

return(L)

}

vrs <- c("ID", "Gruppe", "Geschlecht", "Alter", "Gruppenaustausch", "Strategien")

a <- rn_analys(y = 'Einst.Gehirn_1', covrs = vrs, creatPlots = FALSE)

#****************************************************************************

#   ANALYSIS FUNCTION   ####

#****************************************************************************

#' This function is supposed to include all analysis

#' steps for a specific target variable.

rn_analys <- function(y, covrs=covars, dat=dat_w, m=5, creatPlots=TRUE){

#****************************************************************************

#   PLOT TRAJECTORY   ####

#****************************************************************************

### Colours:

cls <- c('darkblue', 'darkred', 'forestgreen')
```

A randomized controlled trial study of a multimodal intervention vs. cognitive training to foster cognitive and affective health in older adults

Maria Brasser, Sascha Frühholz, Andres R. Schneeberger, Gian G. Ruschetti, Rahel Schaerli, Michèle Häner, Barbara Studer-Luethi

```
if(creatPlots){

### Plot the trajectory of y for each group:

png(filename = paste0('./Figures/', y, '_groupTraj.png'), width = 30,

height = 25, units = 'cm', pointsize = 16, res = 300)

par(mfrow=c(2,2))

par(mar=c(4.5, 4.1, 2.5, 0.1))

### Create reduced Dataset:

d <- dat_l[,c("ID", "Gruppe", "Timepoint", y)]

### Plot each group separately:

L_d <- split(d, f = d$Gruppe)

### ylims:

toadd <- diff(range(d[,y], na.rm = TRUE))*0.1

ymm <- c(min(d[,y], na.rm=TRUE)- toadd, max(d[,y], na.rm = TRUE) + toadd)

### Plot with for loop:

for(i in 1:length(L_d)){

### Plot points:

niceUnivPlot(numVar = L_d[[i]][,y],   # Change the variable here

showMean = FALSE,

violin = FALSE,

lnk.means = cls[i],

catVar = L_d[[i]]$Timepoint,

pairedVar = NULL,

plot.points = TRUE, sigGroup = FALSE,

fxdCol = cls[i], xlab = 'Timepoint', ylab = y, main=paste0('Group ', L_d[[i]]$Gruppe[1]),

lnk.means.lwd = 9, ylim.cust = ymm,

add = ifelse(i==1, FALSE, FALSE))   # Change this to plot all groups together

### Add the trajectories and/or violins:

niceUnivPlot(numVar = L_d[[i]][,y],   # Change the variable here

showMean = FALSE,

violin = FALSE,
```

```
catVar = L_d[[i]]$Timepoint,

pairedVar = L_d[[i]]$ID,

plot.points = FALSE,

fxdCol = mktransp(cls[i], 30),

pairCol = mktransp(cls[i], alpha = 30),

add = TRUE)   # Change this to plot all groups together

}

### Plot all groups together in one figure:

for(i in 1:length(L_d)){

### Plot points:

niceUnivPlot(numVar = L_d[[i]][,y],   # Change the variable here

showMean = FALSE,

violin = FALSE,

lnk.means = cls[i],

catVar = L_d[[i]]$Timepoint,

pairedVar = NULL,

plot.points = TRUE, sigGroup = FALSE,

fxdCol = cls[i], xlab = 'Timepoint', ylab = y, main='All groups together',

lnk.means.lwd = 9, ylim.cust = ymm,

add = ifelse(i==1, FALSE, TRUE))   # Change this to plot all groups together

### Add the trajectories and/or violins:

niceUnivPlot(numVar = L_d[[i]][,y],   # Change the variable here

showMean = FALSE,

violin = FALSE,

catVar = L_d[[i]]$Timepoint,

pairedVar = L_d[[i]]$ID,

plot.points = FALSE,

fxdCol = mktransp(cls[i], 30),

pairCol = mktransp(cls[i], alpha = 30),

add = TRUE)   # Change this to plot all groups together
```

```
}

dev.off()

}

#******************************************************************************

#   NA PLOT AND TABLE   ####

#******************************************************************************

if(creatPlots){

### Create dataset for NA plot:

d <- longToWide(x = dat_l[,c("ID", "Timepoint", y)], colNmStr = '_',

IDvar = 'ID', repColnm = 'Timepoint', repVars = y)

### Generate NA plot:

png(filename = paste0('./Figures/', y, '_NAplot.png'), width = 20,

height = 7, pointsize = 10, units = 'cm', res = 300)

par(mfrow=c(1,1))

par(mar=c(1, 5, 2.5, 2))

niceNaPlot(d, IDvar = 'ID', show_xlab = FALSE)

dev.off()

}

### Create NA table:

d <- dat_l[,c("ID", "Timepoint", y)]

mssTbl <- matrix(NA, nrow = 1, ncol = nlevels(dat_l$Timepoint))

colnames(mssTbl) <- levels(d$Timepoint)

nmss <- NA   # Number of missing

ntt <- nrow(dat_w)   # Number of (potential) data points at each time point

pmss <- NA   # Proportion of missing

for(i in 1:nlevels(d$Timepoint)){

nmss[i] <- sum(is.na(d[as.numeric(d$Timepoint)==i,y]))

pmss[i] <- round(nmss[i]/ntt*100, 1)

mssTbl[i] <- paste0(nmss[i], '/', ntt, ' (', pmss[i], '%)')

}
```

A randomized controlled trial study of a multimodal intervention vs. cognitive training to foster cognitive and affective health in older adults

Maria Brasser, Sascha Frühholz, Andres R. Schneeberger, Gian G. Ruschetti, Rahel Schaerli, Michèle Häner, Barbara Studer-Luethi

```
mssTbl.2 <- cbind(mssTbl, 'Total'= paste0(sum(nmss), '/', nlevels(d$Timepoint)*ntt, ' (', round(100*sum(nmss)/(nlevels(d$Timepoint)*ntt), 1),'%)'))

mssTbl_fin <- noquote(mssTbl.2)

#*****************************************************************************

#   DATA IMPUTATION   ####

#*****************************************************************************

### Impute wide data:

#' ID is not used as predictor in imputation. I also removed

#' Bildung because it only created problems as a (categorial) predictor,

#' due to very low numbers of occurrences of certain levels.

m.out <- mice(dat, m = m, pred=quickpred(dat,

exclude=colnames(dat)[colnames(dat) %in% c("ID", "Bildung")]))

### Collect completed datasets in list:

comp.out <- vector('list', length = m)

for(i in 1:m){

comp.out[[i]] <- complete(m.out, action = i)

}

### Turn each completed data set to long format:

comp.out_l <- lapply(comp.out, FUN = function(x){

d_l <- wideToLong(x, nRep = 3, repColnm = 'Timepoint')

d_l$Strategien <- as.factor(d_l$Strategien)

return(d_l)

})

#*****************************************************************************

#   RUN ANALYSES   ####

#*****************************************************************************

### Define significance level:

apha <- 0.05

### Fit mixed model:

frml <- as.formula(paste0(y, '~ Timepoint * Gruppe + ',
```

```
paste0(covrs[covrs!='ID' & covrs!='Gruppe'],
collapse = '+')))
imp_fit <- lapply(comp.out_l, function(x){
lme(fixed = frml, data = x, random = ~1|ID)
})
### Pool results:
md_res_0 <- summary(pool(imp_fit))
md_res_0$sig <- ifelse(md_res_0$p.value<apha, yes = '*', no = '')
md_res <- md_res_0
#*******************************
### T1 - T2 differences for each group (and difference in effects between groups!):
### Prepare result table:
res_tbl <- matrix(NA, nrow=6, ncol = 5)
rownames(res_tbl) <- c('Gruppe_1', 'Gruppe_2', 'Gruppe_3',
'Gruppe_1 vs. Gruppe_2', 'Gruppe_1 vs. Gruppe_3',
'Gruppe_2 vs. Gruppe_3')
#' Not sure what the easiest way is to create such a table.
#' I will take the somewhat ugly route of refitting the models
#' and extracting the relvant terms.
### First group:
imp_fit <- lapply(comp.out_l, function(x){
lme(fixed = frml, data = x, random = ~1|ID)
})
### Pool results:
res <- summary(pool(imp_fit))
colnames(res_tbl) <- colnames(res)[-1]
### Extract relevant rows:
res_tbl[c("Gruppe_1"),] <- as.numeric(res[res$term=='TimepointT2_', -1])
res_tbl[c("Gruppe_1 vs. Gruppe_2"),] <- as.numeric(res[res$term=='TimepointT2_:Gruppe2', -1])
res_tbl[c("Gruppe_1 vs. Gruppe_3"),] <- as.numeric(res[res$term=='TimepointT2_:Gruppe3', -1])
```

```
### Second group:

imp_fit <- lapply(comp.out_l, function(x){

dd <- x

dd$Gruppe <- factor(dd$Gruppe, levels = c(2, 1, 3))

lme(fixed = frml, data = dd, random = ~1|ID)

})

### Pool results:

res <- summary(pool(imp_fit))

### Extract relevant rows:

res_tbl[c("Gruppe_2"),] <- as.numeric(res[res$term=='TimepointT2_', -1])

res_tbl[c("Gruppe_2 vs. Gruppe_3"),] <- as.numeric(res[res$term=='TimepointT2_:Gruppe3', -1])

### Third group:

imp_fit <- lapply(comp.out_l, function(x){

dd <- x

dd$Gruppe <- factor(dd$Gruppe, levels = c(3, 1, 2))

lme(fixed = frml, data = dd, random = ~1|ID)

})

### Pool results:

res <- summary(pool(imp_fit))

### Extract relevant rows:

res_tbl[c("Gruppe_3"),] <- as.numeric(res[res$term=='TimepointT2_', -1])

### Add significance column:

res_tbl <- as.data.frame(round(res_tbl, digits = 2))

res_tbl$sig <- ifelse(res_tbl$p.value<apha, yes = '*', no = '')

### Extract object:

t1t2_res <- res_tbl

#*******************************

### T2 - T3 differences for each group (and difference in effects between groups!):

### Prepare result table:

res_tbl <- matrix(NA, nrow=6, ncol = 5)
```

```
rownames(res_tbl) <- c('Gruppe_1', 'Gruppe_2', 'Gruppe_3',
'Gruppe_1 vs. Gruppe_2', 'Gruppe_1 vs. Gruppe_3',
'Gruppe_2 vs. Gruppe_3')
### First group:
imp_fit <- lapply(comp.out_l, function(x){
dd <- x
dd$Timepoint <- factor(dd$Timepoint, levels = levels(x$Timepoint)[c(2,1,3)])
lme(fixed = frml, data = dd, random = ~1|ID)
})
### Pool results:
res <- summary(pool(imp_fit))
colnames(res_tbl) <- colnames(res)[-1]
### Extract relevant rows:
res_tbl[c("Gruppe_1"),] <- as.numeric(res[res$term=='TimepointT3_', -1])
res_tbl[c("Gruppe_1 vs. Gruppe_2"),] <- as.numeric(res[res$term=='TimepointT3_:Gruppe2', -1])
res_tbl[c("Gruppe_1 vs. Gruppe_3"),] <- as.numeric(res[res$term=='TimepointT3_:Gruppe3', -1])
### Second group:
imp_fit <- lapply(comp.out_l, function(x){
dd <- x
dd$Gruppe <- factor(dd$Gruppe, levels = c(2, 1, 3))
dd$Timepoint <- factor(dd$Timepoint, levels = levels(x$Timepoint)[c(2,1,3)])
lme(fixed = frml, data = dd, random = ~1|ID)
})
### Pool results:
res <- summary(pool(imp_fit))
### Extract relevant rows:
res_tbl[c("Gruppe_2"),] <- as.numeric(res[res$term=='TimepointT3_', -1])
res_tbl[c("Gruppe_2 vs. Gruppe_3"),] <- as.numeric(res[res$term=='TimepointT3_:Gruppe3', -1])
### Third group:
imp_fit <- lapply(comp.out_l, function(x){
```

A randomized controlled trial study of a multimodal intervention vs. cognitive training to foster cognitive and affective health in older adults

Maria Brasser, Sascha Frühholz, Andres R. Schneeberger, Gian G. Ruschetti, Rahel Schaerli, Michèle Häner, Barbara Studer-Luethi

```
dd <- x

dd$Gruppe <- factor(dd$Gruppe, levels = c(3, 1, 2))

dd$Timepoint <- factor(dd$Timepoint, levels = levels(x$Timepoint)[c(2,1,3)])

lme(fixed = frml, data = dd, random = ~1|ID)

})

### Pool results:

res <- summary(pool(imp_fit))

### Extract relevant rows:

res_tbl[c("Gruppe_3"),] <- as.numeric(res[res$term=='TimepointT3_', -1])

### Add significance column:

res_tbl <- as.data.frame(round(res_tbl, digits = 2))

res_tbl$sig <- ifelse(res_tbl$p.value<apha, yes = '*', no = '')

### Extract object:

t2t3_res <- res_tbl

#*******************************

### T1 - T2 vs. T2 - T3 differences for each group:

#' In order to get this difference I need to run linear hypotheses

#' on the fitted model.

### Prepare result table:

res_tbl <- matrix(NA, nrow=3, ncol = 5)

rownames(res_tbl) <- c('Gruppe_1', 'Gruppe_2', 'Gruppe_3')

### First group:

imp_fit <- lapply(comp.out_l, function(x){

dd <- x

dd$Timepoint <- factor(dd$Timepoint, levels = levels(x$Timepoint)[c(2,1,3)])

lme(fixed = frml, data = dd, random = ~1|ID)

})

### Linear hypothesis:

res <- linearHypothesis(as.mira(imp_fit), "-1*TimepointT1_ - 1*TimepointT3_ = 0")

est <- as.numeric(attributes(res)$value)
```

```
colnames(res_tbl) <- c('Estimate', attributes(res)$names)

res_tbl[c("Gruppe_1"),] <- c(est, res$F, res$`num df`, res$`den df`, res$`Pr(>F)`)

### Second group:

imp_fit <- lapply(comp.out_l, function(x){

dd <- x

dd$Gruppe <- factor(dd$Gruppe, levels = c(2, 1, 3))

dd$Timepoint <- factor(dd$Timepoint, levels = levels(x$Timepoint)[c(2,1,3)])

lme(fixed = frml, data = dd, random = ~1|ID)

})

### Linear hypothesis:

res <- linearHypothesis(as.mira(imp_fit), "-1*TimepointT1_ - 1*TimepointT3_ = 0")

est <- as.numeric(attributes(res)$value)

res_tbl[c("Gruppe_2"),] <- c(est, res$F, res$`num df`, res$`den df`, res$`Pr(>F)`)

### Third group:

imp_fit <- lapply(comp.out_l, function(x){

dd <- x

dd$Gruppe <- factor(dd$Gruppe, levels = c(3, 1, 2))

dd$Timepoint <- factor(dd$Timepoint, levels = levels(x$Timepoint)[c(2,1,3)])

lme(fixed = frml, data = dd, random = ~1|ID)

})

### Linear hypothesis:

res <- linearHypothesis(as.mira(imp_fit), "-1*TimepointT1_ - 1*TimepointT3_ = 0")

est <- as.numeric(attributes(res)$value)

res_tbl[c("Gruppe_3"),] <- c(est, res$F, res$`num df`, res$`den df`, res$`Pr(>F)`)

### Add significance:

res_tbl <- as.data.frame(round(res_tbl, digits = 2))

res_tbl$sig <- ifelse(res_tbl$`Pr(>F)`<apha, yes = '*', no = '')

### Extract object:

t1t2vst2t3_res <- res_tbl

#*******************************************************************************
```

A randomized controlled trial study of a multimodal intervention vs. cognitive training to foster cognitive and affective health in older adults

Maria Brasser, Sascha Frühholz, Andres R. Schneeberger, Gian G. Ruschetti, Rahel Schaerli, Michèle Häner, Barbara Studer-Luethi

```
#   PLOT EFFECTS   ####

#*******************************************************************************

### Generate needed table format for grouped barplot:

effpl <- matrix(NA, nrow=3, ncol=4)

rownames(effpl) <- rownames(t1t2_res)[1:3]

colnames(effpl) <- c('T1T2_Effect', 'T2T3_Effect', 'T1T2_sig', 'T2T3_sig')

### Fill up table:

effpl[,'T1T2_Effect'] <- t1t2_res[1:3, 'estimate']

effpl[,'T2T3_Effect'] <- t2t3_res[1:3, 'estimate']

effpl[,c("T1T2_sig")] <- t1t2_res$p.value[1:3]<apha

effpl[,c("T2T3_sig")] <- t2t3_res$p.value[1:3]<apha

if(creatPlots){

### Start device:

png(filename = paste0('./Figures/', y, '_effectPlot.png'), width = 20,

height = 10, pointsize = 10, units = 'cm', res = 300)

### Generate basic barplot:

yrang <- range(effpl[,1:2])  # Raw y range

toadd0 <- dist(yrang)/10

toadd <- sign(yrang)*toadd0

yll <- yrang+toadd   # ylims

### Take minimally zero as lower ylim:

yll[1] <- min(c(0, yll[1]))

yll[2] <- max(c(0, yll[2]))

bb <- barplot(effpl[,1:2], beside = TRUE, ylim = yll, col=cls)

### Add legend:

legend('bottomleft', legend = rownames(effpl), col = cls, pch=15)

### Add significance stars:

### Group significances:

displ <- abs(toadd/5)[1]

if(sum(effpl[,3:4])>0){   # Only in case there are sig results
```

A randomized controlled trial study of a multimodal intervention vs. cognitive training to foster cognitive and affective health in older adults

Maria Brasser, Sascha Frühholz, Andres R. Schneeberger, Gian G. Ruschetti, Rahel Schaerli, Michèle Häner, Barbara Studer-Luethi

```
### Draw stars:

text(x = bb[effpl[,3:4]==1],

y=(effpl[,1:2][effpl[,3:4]==1]) + sign((effpl[,1:2][effpl[,3:4]==1]))*displ, label='*', cex=2)

}

### Add comparison stars:

tckl <- abs(toadd/10)[1]   # Good ticklength

cmpCd <- combn(1:3, m = 2)   # All comparisons

yy <- max(yll) - 5*tckl   # First height to plot lines

### T1-T2 comparisons:

t1t2C_sig <- t1t2_res[4:6,'p.value']<apha

for(i in 1:length(t1t2C_sig)){   # Iterate through the groups

if(t1t2C_sig[i]){

xx <- bb[,1][cmpCd[,i]]

### Plot lines:

lines(xx, rep(yy, 2))

### Add star:

text(mean(xx), y = yy + tckl/2, labels = '*', cex=2)

### Update height:

yy <- yy + tckl

}

}

### T2-T3 comparisons:

t2t3C_sig <- t2t3_res[4:6,'p.value']<apha

for(i in 1:length(t2t3C_sig)){   # Iterate through the groups

if(t2t3C_sig[i]){

xx <- bb[,2][cmpCd[,i]]

### Plot lines:

lines(xx, rep(yy, 2))

### Add star:

text(mean(xx), y = yy + tckl/2, labels = '*', cex=2)
```

```
### Update height:
yy <- yy + tckl
}
}
dev.off()
}
#*******************************
### Final return object:
L <- list("mssTbl_fin"=mssTbl_fin, "md_res"=md_res,
"t1t2_res"=t1t2_res, "t2t3_res"=t2t3_res,
"t1t2vst2t3_res"=t1t2vst2t3_res, "y"=y, "effpl"=effpl, cols=cls)
return(L)
}
vrs <- c("ID", "Gruppe", "Geschlecht", "Alter", "Gruppenaustausch", "Strategien")
a <- rn_analys(y = 'Einst.Gehirn_1', covrs = vrs, creatPlots = FALSE)
a
str(a)
a
a <- rn_analys(y = 'Einst.Gehirn_1', covrs = vrs, creatPlots = FALSE, m = 30)
a
#********************************************************************************
#   READ IN AND PREPARE DATA   ####
#********************************************************************************
### Read in data:
dat_w <- read.csv('./Data/data_all_281021_YR.csv', stringsAsFactors = TRUE)
### Add corrected digitspan t3 column:
### Add corrected digitspan t3 column:
#' (These values were faulty so Maria sent me
#' the corrected version of just that column. That's why it
### Add corrected digitspan t3 column:
```

A randomized controlled trial study of a multimodal intervention vs. cognitive training to foster cognitive and affective health in older adults

Maria Brasser, Sascha Frühholz, Andres R. Schneeberger, Gian G. Ruschetti, Rahel Schaerli, Michèle Häner, Barbara Studer-Luethi

```
#' (These values were faulty so Maria sent me

#' the corrected version of just that column. That's why it

#' has to be added manually.

dd <- read.csv('./Data/t3Spalte_digitspan_YR.csv')

### Zeros in this column are actually NA:

dd$Digit.Span.GESAMTSCORE[dd$Digit.Span.GESAMTSCORE==0] <- NA

### Add to dataframe

dat_w$T3_Digitspan <- dd$Digit.Span.GESAMTSCORE

### Find empty/faulty cells:

dat_w$T1_Bewegung_frequ[which(dat_w$T1_Bewegung_frequ=='')] <- NA

dat_w$T1_Bewegung_frequ[which(dat_w$T1_Bewegung_frequ=='2h')] <- 120

dat_w$T1_Bewegung_frequ <- as.numeric(dat_w$T1_Bewegung_frequ)

### Turn variables to factors:

dat_w$ID <- factor(dat_w$ID, levels = dat_w$ID, labels = paste0('S', dat_w$ID))

dat_w$Gruppe <- as.factor(dat_w$Gruppe)

dat_w$Geschlecht <- factor(dat_w$Geschlecht, levels = c(1,2), labels = c('m', 'f'))

dat_w$Wohnsit <- as.factor(dat_w$Wohnsit)

dat_w$Bildung <- as.factor(dat_w$Bildung)

dat_w$Ges.zustand <- as.factor(dat_w$Ges.zustand)

source("~/Desktop/PostDoc_Unterlagen/Arbeit/statistischeBeratung/MariaBrasser_Sept2021/Paper/PlotsForPaper/Plots_For_Paper.R")

source("~/Desktop/PostDoc_Unterlagen/Arbeit/statistischeBeratung/MariaBrasser_Sept2021/Paper/PlotsForPaper/Plots_For_Paper.R")

source("~/Desktop/PostDoc_Unterlagen/Arbeit/statistischeBeratung/MariaBrasser_Sept2021/Paper/PlotsForPaper/Plots_For_Paper.R")

getwd()

source("~/Desktop/PostDoc_Unterlagen/Arbeit/statistischeBeratung/MariaBrasser_Sept2021/Paper/PlotsForPaper/Plots_For_Paper.R")

### Libraries and data (I source the analysis script):

source('../../Report/statBeratung_Maria_Brasser.R')

ls()
```

A randomized controlled trial study of a multimodal intervention vs. cognitive training to foster cognitive and affective health in older adults

Maria Brasser, Sascha Frühholz, Andres R. Schneeberger, Gian G. Ruschetti, Rahel Schaerli, Michèle Häner, Barbara Studer-Luethi

```
vrs <- c("ID", "Gruppe", "Geschlecht", "Alter", "Gruppenaustausch", "Strategien")

a <- rn_analys(y = 'Einst.Gehirn_1', covrs = vrs, creatPlots = FALSE, m = 30)

a

beepr::beep()

a

getwd()

source("~/Desktop/PostDoc_Unterlagen/Arbeit/statistischeBeratung/MariaBrasser_Sept2021/Report/statBeratung_Maria_Brasser.R")

getwd()

setwd("/home/yrothacher/Desktop/PostDoc_Unterlagen/Arbeit/statistischeBeratung/MariaBrasser_Sept2021/Report")
```
